# Supplementary material for: Dynamic force measurements on swimming Chlamydomonas cells using micropipette force sensors
Source: J R Soc Interface. 2020 Jan 15;17(162):20190580. doi: 10.1098/rsif.2019.0580 (PMC7014799; doi:10.1098/rsif.2019.0580)
Supplement: Supplementary Figures [file rsif20190580supp1.pdf]

Supplementary Figures 1 to 6 for Dynamic Force Measurements on Swimming *Chlamydomonas* Cells using Micropipette Force Sensors published in Journal of the Royal Society Interface

Thomas J. Böddeker,[1] Stefan Karpitschka,[1] Christian T. Kreis,[1] Quentin Magdelaine,[1] and Oliver Bäumchen[1]

[1] *Max Planck Institute for Dynamics and Self-Organization (MPIDS), Am Faßberg 17, D-37077 Göttingen, Germany*

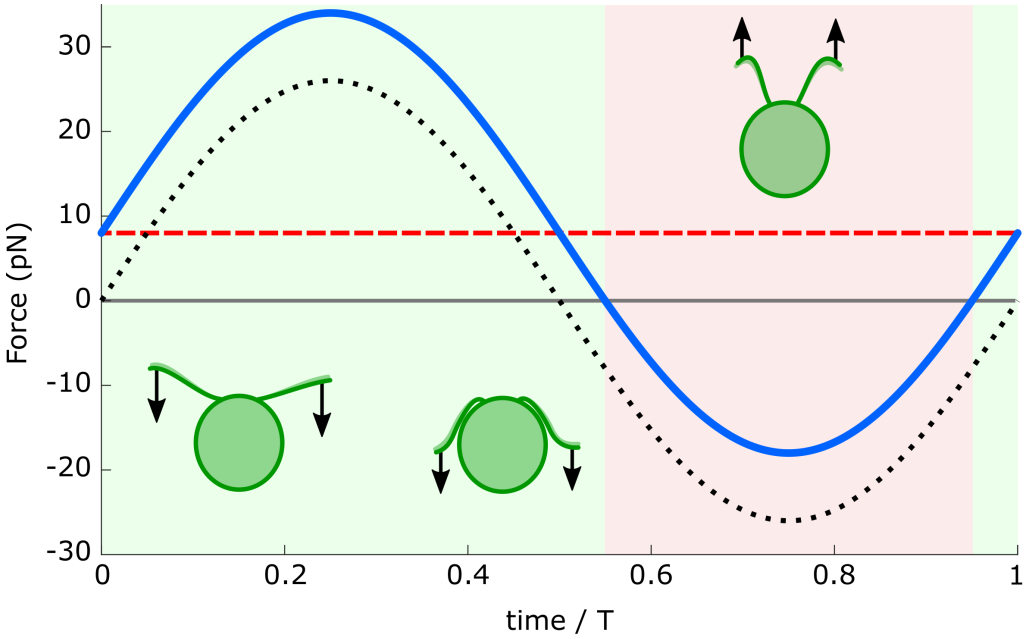

Supplementary Figure 1. Minimal model of the instantaneous forcing of the flagella. The instantaneous forcing of the flagella (solid blue line) has two components. The constant offset (dashed red line) leading to net propulsion and an oscillatory forcing (dotted black line) ensuring ongoing propulsion. The instantaneous forcing is the sum of the oscillatory forcing and the offset, resulting in an asymmetry between propulsive (positive instantaneous forcing, shaded in green) and recovery stroke (negative instantaneous forcing, shaded in red). In Fourier space, the constant mean forcing leads to a peak at zero frequency, the oscillatory forcing leads to a peak or signature at the beating frequency. The sketches of the alga indicate corresponding flagella configurations, the arrows show the instantaneous velocity of the flagella.

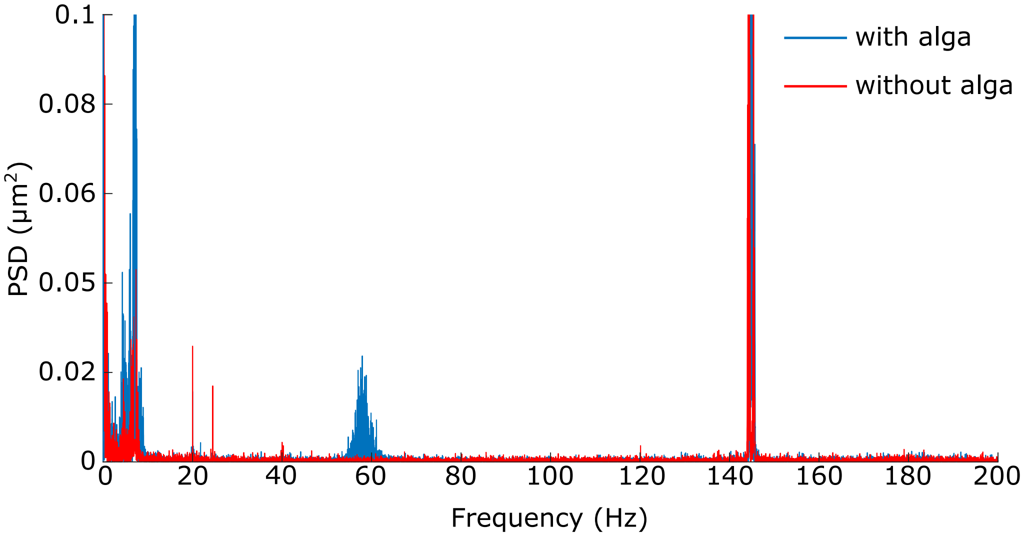

Supplementary Figure 2. Full power spectrum of the data presented in Figure 2. Both signals with and without alga show significant environmental noise at low frequencies below 10 Hz. The large and practically identical signature in both spectra just above 140 Hz originates from the fan of the high-speed camera. The range of the y-axis has been adjusted such that the signal originating from the alga is still visible.

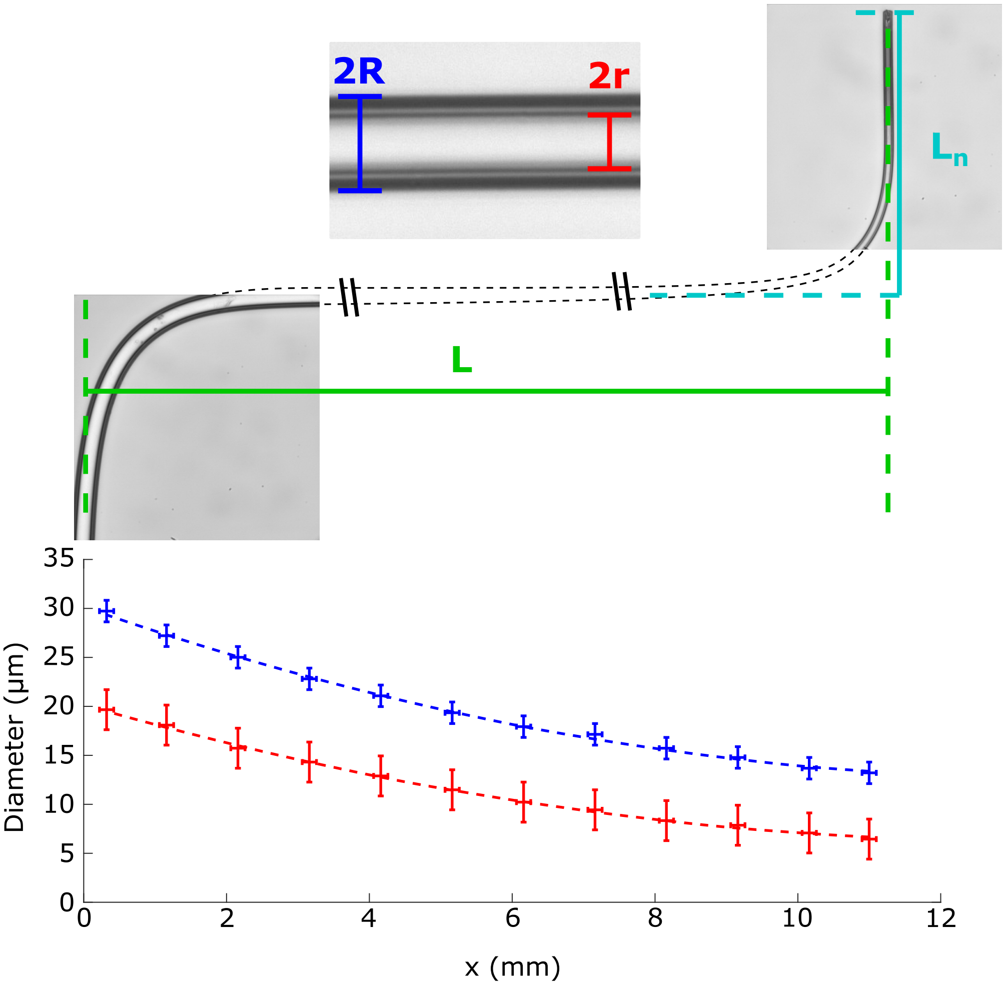

Supplementary Figure 3. Precise measurement of the micropipette's geometric parameters. Inner and outer diameter of the micropipette cantilever $2r$ and $2R$ were measured based on optical micrographs of the micropipette (typical error of $2\,\mu\mathrm{m}$) taken at equidistant intervals along the micropipette cantilever. The cantilever length L was measured based on the movement of the micromanipulator; the nozzle length L_n was directly obtained from micrographs. To extract the precise shape of the micropipette, we fit a second order polynomial to the acquired data for the inner and outer diameter.

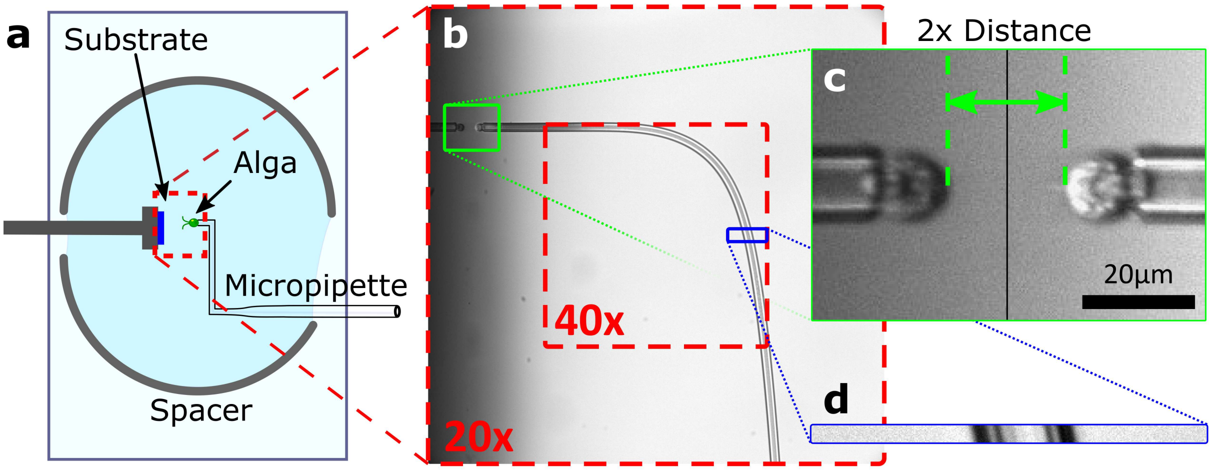

Supplementary Figure 4. Experimental setup and orientation of the cell in the vicinity of a solid/liquid interface. **a** Schematic of the liquid cell and orientation of the substrate and the micropipette force sensor. **b** Micrograph of a cell held by a micropipette sensor in the vicinity of the silicon substrate. **c** Close-up of the cell and its reflection on the substrate. The distance of the cell to the interface is determined as half the distance from the base of the flagella to the base of the flagella in the reflection of the cell. **d** Close-up of the recorded pipette cross-section used to extract the deflection (at 40x).

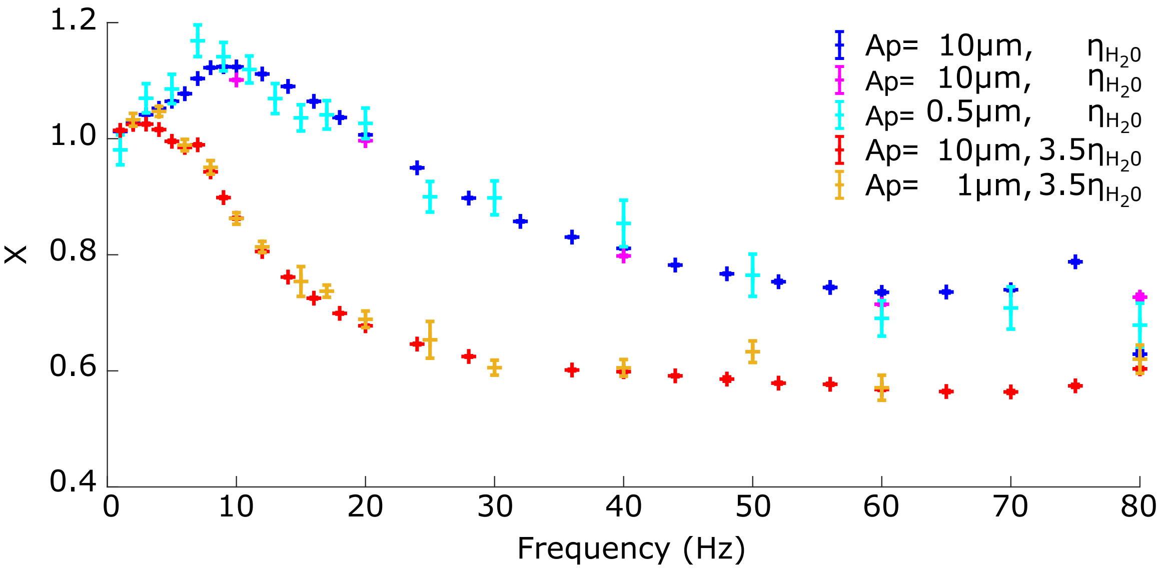

Supplementary Figure 5. Validation of the experimental dynamic calibration method to measure the micropipette force sensor's frequency response $\chi = A/A_{\mathrm{b}}$. Variation of the amplitude of the external actuation of the pipette base A_{b} did not show any significant effect on the frequency response of the cantilever. Upon increasing the viscosity of the surrounding liquid to 3.5 times the viscosity η of water (using a water-glycerol mixture), the damping of the pipette increases as expected for increased hydrodynamic drag.

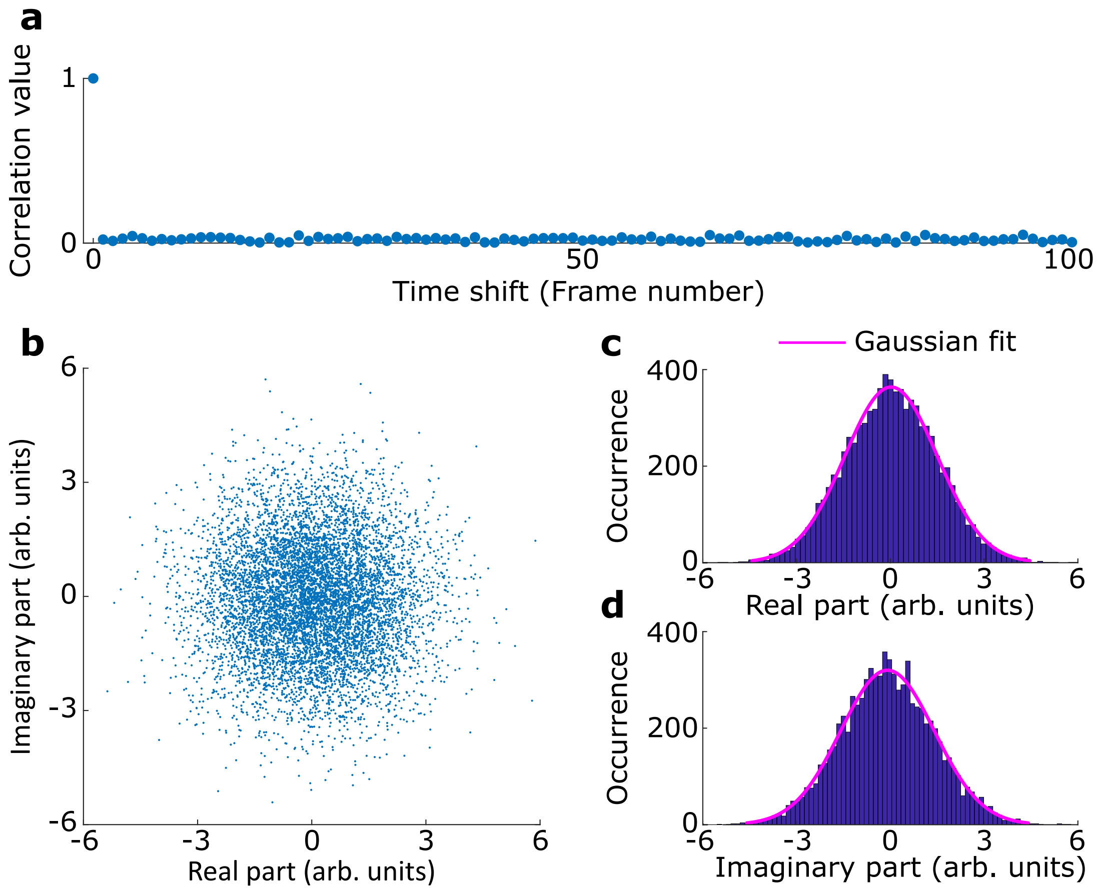

Supplementary Figure 6. Noise analysis of the power spectrum obtained without a cell attached to the force sensor (red signal in **Figure 2** and **Supplementary Figure 2**). **a** Correlation of the noise in the range of 30 to 130 Hz calculated from the inverse Fourier transform of the power spectrum following the Wiener-Khinchin Theorem. The noise is found to be uncorrelated in time. **b** Fourier coefficients corresponding to the same power spectrum in the range of 30 to 130 Hz. **c** Histogram of the real part of the scatter plot shown in **(b)**. The Gaussian fit yields a mean of 0.023 ± 0.033 with a variance of 1.482 ± 0.023. **d** Histogram of the imaginary part of the scatter plot shown in **(b)**. The Gaussian fit yields a mean of -0.092 ± 0.034 with a variance of 1.501 ± 0.023.
